# Fluctuating numbers of circulating tumor cells in cancer patients and the meaning of zero counts

**DOI:** 10.18632/oncotarget.26850

**Published:** 2019-04-12

**Authors:** Nicola Aceto

**Affiliations:** Department of Biomedicine, Cancer Metastasis Laboratory, University of Basel, University Hospital Basel, Basel, Switzerland

**Keywords:** circulating tumor cells, metastasis, denosumab

Circulating tumor cells (CTCs) are cancer cells in transit through the bloodstream, providing a minimally-invasive source of neoplastic material for molecular and phenotypic analysis, as well as an opportunity to monitor disease progression [1]. CTC enumeration in patients with metastatic breast cancer has clearly revealed their correlation to a poorer prognosis in large patient cohorts, with patients having ≥ 5 CTCs per 7.5 ml of peripheral blood being characterized by a shorter median overall survival compared to patients with < 5 CTCs in the same volume of blood [2, 3]. Additionally, several clinical studies have highlighted the prospect to monitor minimal residual disease through CTC analysis [1], while others have started to dissect CTC biology, revealing interesting insights into the metastatic process. These include the assessment of the metastatic potential of single and clustered CTCs, DNA methylation dynamics of CTCs within the bloodstream with an impact on their metastasis-seeding ability, as well as heterotypic cell binding of CTCs to immune cells, a mechanism whereby immune cells favor the proliferative ability of CTCs and their metastasis-forming capability [4–7]. Together, these results have important implications for disease monitoring, treatment and target identification. Yet, the detection of CTCs in peripheral blood specimens of cancer patients remains largely unpredictable, even in patients with advanced disease, representing a challenge for CTC-related investigations and a potential obstacle for clinical studies that rely on CTCs as biomarkers.

We have recently asked whether the detection of CTCs in breast cancer patients could be influenced by clinico-pathological features [8]. One of the main motivations for addressing this question was the observed fluctuations in CTC numbers in patients with comparable disease status (e.g. progressive disease), overall burden and metastatic profile, suggesting that CTC shedding rates are unequal across patients and even within individual patients that are sampled longitudinally, and that possibly, certain clinical features could “accidentally” influence cancer spread. On the other hand, clear correlatives between CTC abundance and patient prognosis have been demonstrated in large clinical studies [2, 3], suggesting a logic connection between CTC release and tumor aggressiveness. Like many other independent prognostic factors though, their presence or absence (or the lack of their detection) in a given patient does not provide certainty of a pre-defined disease outcome, but rather represents the likelihood of a given prognosis. In our study, we find that treatment with the monoclonal antibody Denosumab, a RANKL inhibitor, correlates with the absence of CTCs from the peripheral circulation of breast cancer patients with bone metastasis [8]. This correlation will require validation in prospective patient cohorts where additional clinical endpoints are evaluated, as well as further experimental evidence to dissect the role of Denosumab in CTC generation. For instance, our study did not address whether the effect of Denosumab was mediated through inhibition of RANKL within the bone (i.e. preventing the maturation of preosteoclasts into osteoclasts) or on cancer cells themselves (previously shown to rely on the RANK/RANKL axis in some cases) or both. The investigation of the mechanism of action of Denosumab in the context of CTC generation might be relevant for shedding light into mechanisms that promote CTC intravasation. More generally however, our study suggests that CTC shedding from cancerous lesions might be influenced by tumor-extrinsic factors, including treatment history of the patient but also, potentially, by factors that are yet to be characterized. A better understanding of these phenomena is fundamental towards the identification of metastasis-preventing therapies.

While patients with < 5 CTCs per 7.5ml of peripheral blood are generally characterized by a better prognosis compared to patients with higher counts [2, 3], a proportion of these will nevertheless progress very rapidly, i.e. at a similar rate compared to patients with elevated CTC numbers. In this context, it is important to realize that patients in whom CTCs are not detected in the peripheral circulation at a given timepoint might not be categorically catalogued as CTC-free. For instance, it is unclear whether CTC shedding from cancerous lesions is a constant process or a phenomenon that occurs in “waves”, whereby CTCs are released upon particular stimuli. To this end, longitudinal CTC monitoring in large patient cohorts might help understanding the dynamics of CTC release and to further stratify patients accordingly to their CTC release pattern. Secondly, the absence of CTCs from the peripheral circulation does not exclude that CTCs are released from a growing tumor lesion and immediately trapped within the first (i.e. downstream of the tumor) capillary bed. This scenario would be consistent with a high ability of CTCs to spread to distant sites and adhere to endothelial cells in capillary beds before reaching the periphery, possibly leading to the establishment of new metastases. Previous studies have indeed reported that arterial blood contains more CTCs compared to the venous circulation in patients [9], suggesting that alternative phlebotomy locations might reveal unforeseen CTC patterns and allow CTC isolation before their entrapment within distant sites. Lastly, in the metastatic setting, it is also unclear whether growing metastases in different organs are characterized by different CTC shedding capabilities. It is not unlikely that certain metastatic sites might be less prone to favor CTC intravasation compared to others, almost independently of disease progression status. Studies where blood specimens from patients with defined metastatic patterns are interrogated will help to identify organ-specific features with an impact on CTC release.

In summary, much still needs to be understood regarding CTC biology, shedding patterns and circulation dynamics in several cancer types. Further studies may require creative thinking and unconventional experimental settings to resolve these aspects, aiming at taking full advantage of minimally-invasive blood sampling for cancer detection, patient stratification and treatment.
